# Polyamide 6 as a Liner Material for Type IV Hydrogen Storage Cylinders: Performance Challenges and Modification Strategies

**DOI:** 10.3390/polym17131848

**Published:** 2025-07-01

**Authors:** Wenyan Wang, Guanxi Zhao, Xiao Ma, Dengxun Ren, Min Nie, Rui Han

**Affiliations:** 1 Sichuan Special Equipment Inspection Institute, Technology Innovation Center of Hydrogen Storage-Transportation and Fueling Equipments, State Administration for Market Regulation, Chengdu 610000, China; wwyandmmy@163.com (W.W.); my6900336@163.com (G.Z.); 2 Key Laboratory of Materials and Surface Technology (Ministry of Education), School of Materials Science and Engineering, Engineering Research Center of Intelligent Air-Ground Integration Vehicle and Control, Xihua University, Chengdu 610039, China; rendenxun2008@xhu.edu.cn; 3 China Southwest Architectural Design and Research Institute Corp., Ltd., Chengdu 610041, China; mx18183274820@163.com; 4 State Key Laboratory of Polymer Materials Engineering, Polymer Research Institute of Sichuan University, Chengdu 610065, China

**Keywords:** type IV hydrogen storage cylinder, polyamide 6, hydrogen barrier properties, mechanical properties

## Abstract

Type IV hydrogen storage cylinders are pivotal for high-pressure hydrogen storage and transportation, offering advantages such as lightweight design, high hydrogen storage density, and cost efficiency. Polyamide 6 (PA6) has emerged as a promising liner material due to its excellent mechanical strength, chemical resistance, and gas barrier properties. However, challenges remain, including high hydrogen permeability and insufficient mechanical performance under extreme temperature and pressure conditions. This review systematically summarizes recent advances in modification strategies to enhance PA6’s suitability for Type IV hydrogen storage cylinders. Incorporating nanofillers (e.g., graphene, montmorillonite, and carbon nanotubes) significantly reduces hydrogen permeability. In situ polymerization and polymer blending techniques improve toughness and interfacial adhesion (e.g., ternary blends achieve a special increase in impact strength). Multiscale structural design (e.g., biaxial stretching) and process optimization further enhance PA6’s overall performance. Future research should focus on interdisciplinary innovation, standardized testing protocols, and industry–academia collaboration to accelerate the commercialization of PA6-based composites for hydrogen storage applications. This review provides theoretical insights and engineering guidelines for developing high-performance liner materials.

## 1. Introduction

**Figure 1:**
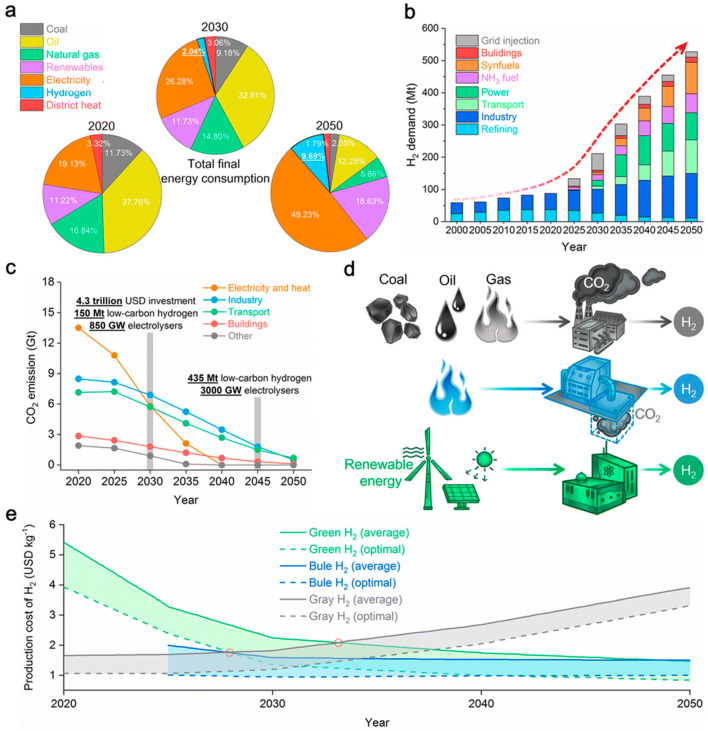
The future outlook for hydrogen energy development: (**a**) Energy mix percentages by source (2020–2050), (**b**) Hydrogen allocation by sector (capacity units), (**c**) Hydrogen infrastructure scale-up targets, (**d**) CO_2_ emissions vs. renewable energy trends, (**e**) Hydrogen production cost evolution by type [7].

Global warming and the fossil fuel crisis are accelerating the transition to cleaner energy sources [1,2]. Hydrogen, as a cleaner energy with zero carbon emissions and a high energy density of 33.6 kWh/kg, is seen as a key solution to both energy and environmental problems [3,4,5]. However, one of the biggest challenges in using hydrogen on a large scale is how to store and transport it. Because hydrogen molecules are very small and their boiling point is extremely low (−253 °C), their energy density by volume is only about one-third that of natural gas, leading to energy losses during transportation using traditional methods [6,7]. As hydrogen energy systems continue to develop (Figure 1) [7], effective storage and transportation methods remain key technical considerations. There are three main ways to store hydrogen: as a high-pressure gas, as a low-temperature liquid, or through solid-state adsorption [8,9,10]. Among them, high-pressure gas storage is the most commonly used in vehicles because it is mature and allows fast charging and discharging [11,12]. The hydrogen storage cylinders are the key part of this system, and their performance directly affects the safety and cost of hydrogen storage and transport.

**Figure 2:**
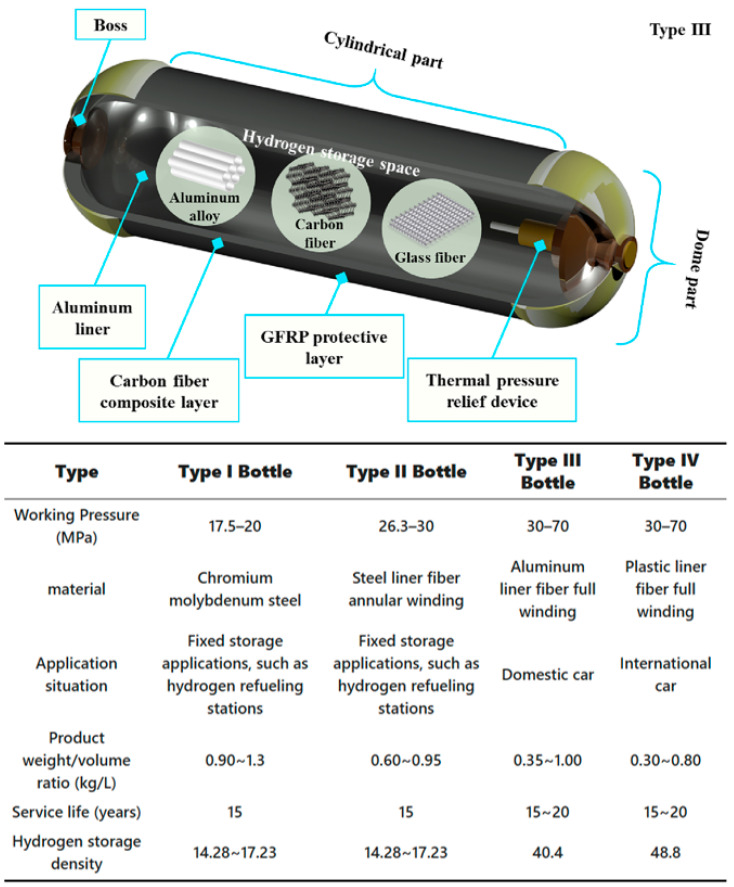
Development and structural composition of hydrogen storage cylinders [11,16].

As the core equipment for hydrogen storage and transportation, hydrogen storage cylinders play a critical role in the commercialization of hydrogen energy. The technology behind these cylinders has gone through four major upgrades, especially in the materials used (Figure 2). The earliest Type I cylinders were made entirely of metal, followed by Type II cylinders, which combined a metal liner with partial fiber reinforcement [13,14]. Both types are mainly suitable for low-pressure applications below 35 MPa. Currently, the dominant technologies are Type III and Type IV cylinders. Type III cylinders use a metal liner fully wrapped with carbon fiber (Figure 2), while Type IV cylinders represent a more advanced design by using a plastic liner fully wrapped with carbon fiber [14,15,16]. These innovations have enabled the practical use of hydrogen storage at pressures up to 70 MPa. Among them, Type IV cylinders offer several clear advantages due to the use of polymer liners instead of metal. These include reduced weight, higher hydrogen storage density, lower cost thanks to reduced carbon fiber usage, and improved manufacturing efficiency [17,18]. Because of these benefits, Type IV cylinders have shown strong potential in high-pressure hydrogen storage and have become a major focus for research and industrial development. In particular, the performance of the plastic liner in Type IV cylinders is key to their large-scale adoption and practical application.

## 2. The Liner in Type IV Hydrogen Storage Cylinders

**Figure 3:**
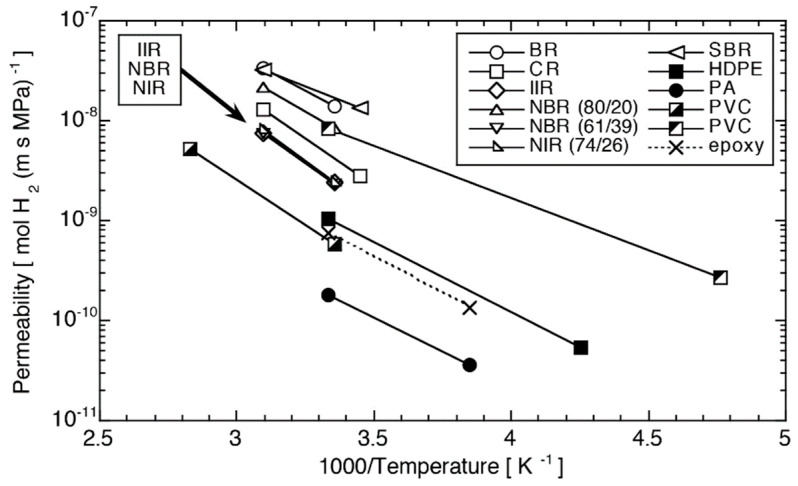
Hydrogen permeability of various polymer materials [21].

The polymer liner in Type IV hydrogen storage cylinders plays a vital role in ensuring the safety, efficiency, and reliability of the entire hydrogen storage system [19,20]. As the inner layer, the polymer is responsible for three key functions. First, it provides an effective seal to prevent the leakage of high-pressure hydrogen gas. Second, it offers structural support for the outer carbon fiber wrapping, helping the cylinder maintain its shape and strength under pressure. Third, it helps absorb and distribute external forces, reducing the risk of damage to the composite structure. However, polymer liner materials still face significant technical challenges. Because hydrogen molecules can easily penetrate and diffuse through materials, making it essential for the liner to have excellent hydrogen barrier properties. In addition, the material must maintain stable mechanical performance under extreme temperature conditions, ranging from −40 °C to 85 °C. It needs to be strong enough to withstand high pressure while also remaining tough and flexible enough to avoid brittle failure. At present, the most commonly studied and used liner materials include high-density polyethylene (HDPE) and polyamide (PA), both of which offer promising properties for hydrogen containment [19,21,22]. According to the research, HDPE’s hydrogen diffusivity is 2 × 10^−9^·m^2^·s^−1^ at 50 °C, while PA’s hydrogen diffusivity is an order of magnitude lower at 4× 10^−10^·m^2^·s^−1^ [19]. Moreover, Smith et al. conducted a systematic study on three commercially polymer liner materials: injection-molded HDPE, rotationally molded HDPE, and extruded polyamide 6 (PA6) [22]. Under high pressure (13.4 MPa) and extreme temperature conditions ranging from −40 °C to 85 °C, they performed 1500 thermal cycles to evaluate the materials’ durability and hydrogen permeability. The results show that, in tests measuring how permeability changes with temperature (Figure 3), PA6 performs similarly to extruded HDPE and significantly better than injection-molded HDPE. In hydrogen permeability tests under different pressures (Figure 3), PA6 consistently outperforms HDPE in terms of gas barrier performance. Overall, PA-based materials demonstrate superior properties in mechanical strength, hydrogen barrier property, and thermal stability, making them one of the most promising candidates for industrial use as liner materials.

Based on current research, this review systematically summarizes the key properties of PA6 as a liner material for Type IV hydrogen cylinders, aiming to enhance their safety and commercial application. The analysis focuses on PA6’s hydrogen barrier performance and mechanical behavior. By evaluating material modifications, structural designs, and performance testing methods, this work aims to provide scientific and engineering insights for developing safer polymer liners.

## 3. Analysis of the Intrinsic Properties of PA6

**Figure 4:**
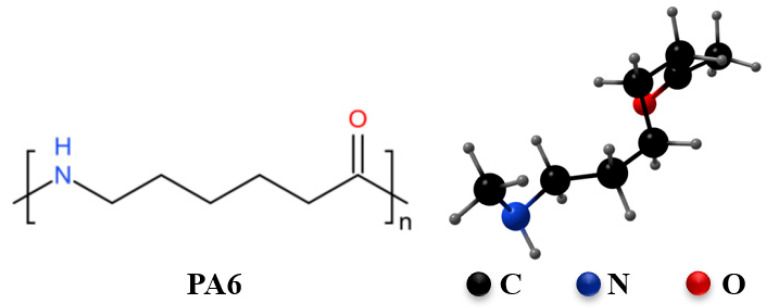
The molecular structural formula of PA6.

PA6 is a semi-crystalline thermoplastic polymer, with its molecular backbone composed of repeating amide bonds (–CO–NH–) and methylene segments [23,24], as illustrated in Figure 4. This molecular structure gives PA6 excellent mechanical strength, chemical resistance, and gas barrier properties. The polar amide bonds enhance intermolecular hydrogen bonding, resulting in high tensile strength (around 80 MPa) and modulus [25]. Additionally, the crystallinity of PA6—typically ranging from 30% to 50%—can be adjusted through processing conditions [26], allowing further improvement of its gas barrier performance. This is particularly important in high-pressure hydrogen environments, where low permeability is critical. Compared to other polyamides such as PA12 or PA11, PA6 achieves a better balance between cost, processability, and overall performance [27,28].

Despite its promising properties, the PA6, used as a liner material in hydrogen storage cylinders, still faces challenges in both hydrogen barrier performance and mechanical properties. In terms of hydrogen barrier performance, PA6 outperforms polyolefins due to its crystalline structure and polar amide bonds [28,29]. However, hydrogen molecules can still permeate through the polymer’s amorphous regions, particularly under high-pressure conditions. Moreover, conventional methods for increasing crystallinity to improve barrier properties often come at the cost of reduced toughness, which can compromise the material’s overall durability. On the mechanical side, the strong hydrogen bonding network in PA6 provides high tensile strength, but it also limits the ability to dissipate energy, particularly under low temperatures or dynamic loading conditions [30,31]. This reduced energy absorption may lead to brittleness and potential failure during long-term service or in crash scenarios. As a result, current research is largely focused on enhancing the hydrogen barrier properties and the mechanical performance of PA6 to improve its safety and reliability as a liner material in Type IV hydrogen storage cylinders.

## 4. Hydrogen Barrier Properties of PA6

### 4.1. Gas Permeation Mechanism

**Figure 5:**
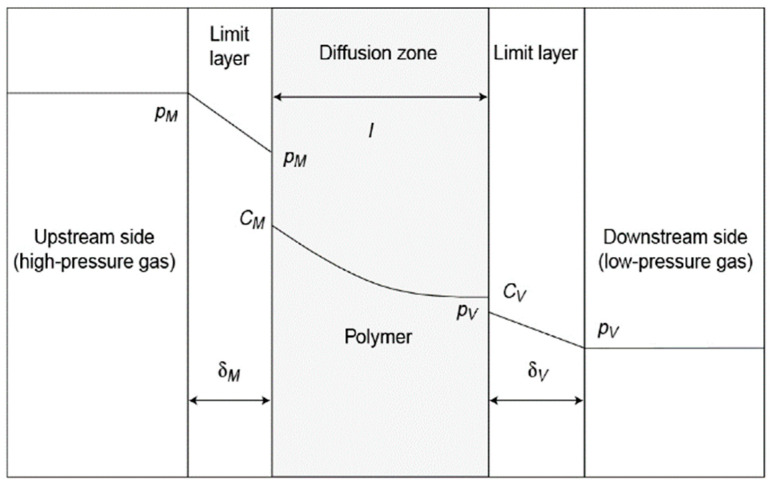
The diffusion process of gas in the polymer [21,32].

Gas permeation through polymers follows a classical solution–diffusion mechanism [21,32]. As illustrated in Figure 5, the high-pressure gas first diffuses into the upstream side of the material, forming a boundary layer. Due to the chemical affinity between the gas and the polymer’s upstream interface, gas molecules accumulate and dissolve at the polymer surface. Subsequently, the dissolved gas molecules continue to diffuse through the polymer matrix until they reach the downstream interface. Finally, the gas desorbs from the polymer at the low-pressure side.

The gas permeability of a polymer reflects its ability to allow gas molecules to pass through it. This permeability coefficient (P) is determined by the combined effect of the diffusion coefficient (D) and the solubility coefficient (S), as described by Equation (1) [33]:

(1) P=S×D

The S indicates how readily gas molecules can dissolve into the polymer and is mainly affected by the chemical affinity between the gas and the polymer chains [21]. Because hydrogen has low solubility in polymers, its movement through materials like PA6 mainly depends on how easily it can diffuse. The D reflects how quickly gas molecules can move through the polymer matrix, which is largely influenced by the polymer’s microstructure, such as free volume and chain mobility [34]. In crystalline areas of PA6, the molecules are tightly packed, which creates barriers that slow down hydrogen. However, in less ordered regions—like amorphous zones or areas with defects—hydrogen can pass through more easily. Besides, small defects inside the polymer, such as cracks or pores, can greatly increase the rate at which hydrogen passes through [35]. In PA6, the hydrogen bonds between amide groups in the polymer chain help reduce free space, which naturally improves its ability to block hydrogen. To reduce hydrogen permeability further, techniques like biaxial stretching or adding fibers can help align the polymer chains, making the path for hydrogen longer and harder to pass through [36,37]. Environmental factors, like high temperature and pressure, also affect the hydrogen permeability of PA6 [38]. To solve these problems, different strategies are studied, like improving the crystalline structure and adding nanomaterials, to make PA6 more resistant to hydrogen, even under high pressure and across a wide range of temperatures.

### 4.2. Gas Barrier Enhancement Technologies

**Figure 6:**
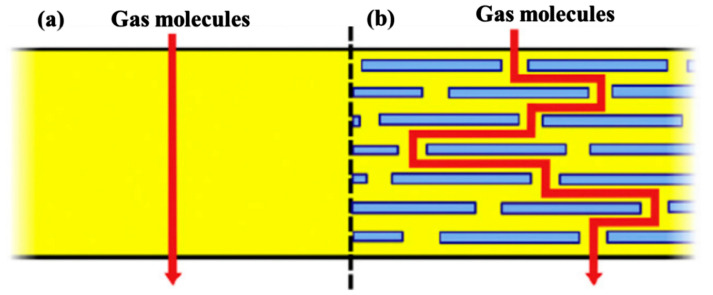
Schematic diagram of gas permeation through polymers without (**a**) and with (**b**) nanofillers [40].

Studies have shown that adding nanofillers—such as montmorillonite (MMT), carbon nanotubes, or graphene—into polymer matrices can significantly improve their gas barrier properties [39,40]. This enhancement mainly comes from the unique physical characteristics of nanomaterials. Their extremely high surface area and impermeable nature can change how gas molecules move through the material. As shown in Figure 6, instead of passing straight through, the gas molecules are forced to navigate around these obstacles, which effectively lengthens and complicates the diffusion path [40]. As a result, the overall gas permeability of the polymer is greatly reduced.

#### 4.2.1. Graphene and Its Derivatives

**Figure 7:**
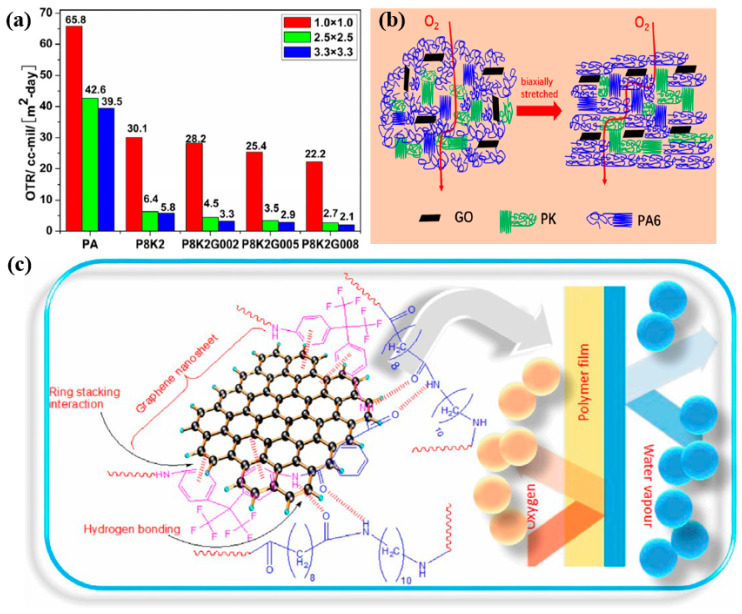
The oxygen permeability of PA6 composites (**a**); schematic diagram of gas barrier mechanisms in PA6 composites (**b**); and the barrier effect of functional graphene (**c**) [43,45].

Graphene is a single-layer structure made up of sp^2^-hybridized carbon atoms arranged in a hexagonal lattice [41,42]. It is considered the basic structural unit of various carbon-based materials such as graphene nanoplatelets, carbon nanotubes (CNTs), and carbon nanofibers (CNFs). These materials not only exhibit excellent mechanical, electrical, and thermal properties but also possess a relatively high aspect ratio, which significantly extends the diffusion pathway for gas molecules. Because of this, graphene and its derivatives have become promising additives for improving the gas barrier performance of polymers. For example, Lin et al. prepared PA6/polyketone (PK)/graphene oxide (GO) composite films using a melt blending and biaxial stretching process [43]. When the mass fractions of PK and GO are 20% and 0.08%, respectively, the oxygen permeability drops by 94.7% (Figure 7a). This improvement is attributed to the high alignment of GO nanosheets during biaxial stretching, which greatly increases the tortuosity of gas diffusion paths (Figure 7b). Moreover, the presence of GO also induces higher crystallinity in PA6, reducing the number of amorphous regions available for gas diffusion. Similarly, Raine et al. introduced graphene nanoplatelets into PA6 laminates, and under supercritical conditions, the CO_2_ permeability was reduced by an order of magnitude, while H_2_S permeability dropped below detectable limits [44]. This is primarily due to the dense, layered structure of graphene, which effectively blocks the gas molecules. Kausar prepared graphene by in situ chemical reduction of graphene oxide and incorporated it into a novel blend of fluorinated polyamide (FPA) and polyamide 1010 (PA1010), creating a multifunctional nanocomposite [45]. Compared to the unmodified blend, the coated nanocomposites show excellent barrier performance against O_2_ and H_2_O because of the barrier effect of functional graphene (Figure 7c). Molecular dynamics simulations show that when the graphene content in PA6 reaches 5 wt%, the permeability coefficient drops to 2.44 × 10^−13^ cm^3^·cm/(cm^2^·s·Pa), a reduction of 22–24% compared to pure PA6 [37]. However, increases in temperature and pressure are found to negatively impact the hydrogen barrier performance of the PA6 composites. To further enhance both gas barrier and mechanical properties, multi-walled carbon nanotubes (MWCNTs) and amino-functionalized MWCNTs (MWCNTs-NH_2_) were added into PA6 [46]. Simulated material models reveal that, at various temperatures, the addition of CNTs reduces the free volume fraction, solubility coefficient, and diffusion coefficient of the polymer [46]. These findings are confirmed by oxygen permeability experiments, which show that the permeability of nanocomposites is reduced by approximately 90% compared to neat PA6—consistent with the simulation results. These findings demonstrate the strong potential of carbon nanomaterials in enhancing the gas barrier performance of PA6 for advanced hydrogen storage applications.

#### 4.2.2. Inorganic Nanocomposites

**Figure 8:**
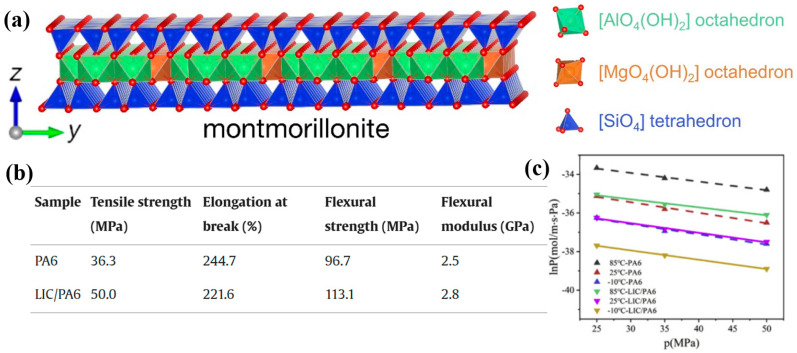
MMT structural diagram (**a**); the mechanical properties table (**b**); and hydrogen permeability (**c**) coefficients of LIC/PA6 composites [49,51].

Montmorillonite (MMT) is a typical layered silicate clay mineral that is widely used in composite materials due to its unique structural characteristics [47,48,49]. Its crystal structure features a classic “sandwich-like” layered arrangement (see Figure 8a), consisting of one aluminum (or magnesium) octahedral sheet between two silicon–oxygen tetrahedral sheets [48,49]. The surfaces of the layers carry permanent negative charges, and the interlayer spaces are held together by electrostatic forces. MMT also exhibits a high aspect ratio, which makes it an excellent functional filler for enhancing the performance of polymer composites.

Due to its low cost and unique structural features, MMT has been widely studied for improving gas barrier properties of polymers. Ito et al. prepared PA6/MMT composites and tested their oxygen permeability at 65 °C, finding that the MMT-filled composites exhibited significantly lower oxygen permeability compared to pure PA6 [50]. Similarly, layered inorganic compounds (LICs) have been used to modify PA6, resulting in enhanced thermal and processing properties [51]. Compared to pure PA6, LIC/PA6 composites demonstrate improvements in tensile strength, flexural strength, and flexural modulus by 36%, 17%, and 12%, respectively (Figure 8b). More importantly, the hydrogen permeability of LIC/PA6 composites decreases by three to five times (Figure 8c), meeting the requirements for hydrogen storage applications. When inorganic fillers are uniformly dispersed in the PA6 matrix, they create a dense physical barrier network that forces gas molecules to follow a more tortuous diffusion path, greatly extending the permeation time. Additionally, the fillers restrict polymer chain mobility and reduce the free volume within the material. This limits both gas dissolution and diffusion. Furthermore, these fillers can act as heterogeneous nucleation sites, promoting crystallinity in PA6 and forming a more ordered crystalline structure that further blocks gas permeation. These synergistic effects enable PA6 composites to exhibit significantly improved gas barrier performance.

The interfacial characteristics between nanofillers and polymer matrix are found to substantially influence gas diffusion behavior. The interfacial adhesion strength modulates diffusion kinetics by altering the geometric tortuosity of gas permeation pathways, while the chain confinement effect in the interfacial region induces conformational rearrangement and suppressed segmental mobility [52,53]. This dual mechanism ultimately leads to a systematic reduction in the diffusion of dissolved gases [52,53,54]. Such interfacial effects become particularly pronounced in nanocomposites containing high-specific-surface-area fillers due to the increased interface-to-volume ratio. Actually, processing techniques critically influence the dispersion and orientation of nanofillers. For instance, biaxial stretching aligns graphene sheets along the stretching direction, forming barrier layers perpendicular to the gas diffusion path and significantly increasing the tortuosity of the diffusion route [55]. The orientation distribution of fillers (e.g., MMT and graphene) within a polymer matrix significantly impacts its barrier properties. When fillers are aligned perpendicular to the permeation direction of small molecules, they create maximally tortuous diffusion pathways, dramatically enhancing barrier efficiency. By precisely controlling orientation (e.g., via biaxial stretching), the researcher can maximize tortuosity and achieve reduction in permeability, offering a scalable strategy for high-barrier nanocomposite development.

## 5. Research on the Mechanical Properties of Polyamide 6

### 5.1. Mechanical Failure of PA 6

The mechanical failure mechanism of PA6 used as the liner material in Type IV hydrogen storage cylinders primarily stems from its long-term performance degradation under cyclic high-pressure loading and hydrogen exposure [56]. During repeated hydrogen charging and discharging cycles at high pressures (typically 35–70 MPa), PA6 undergoes hydrogen-induced plasticization, which weakens intermolecular forces and gradually reduces the material’s stiffness and strength. Simultaneously, the permeation of high-pressure hydrogen creates localized internal pressure zones within the material, triggering the initiation and propagation of microcracks—particularly at the interfaces between crystalline and amorphous regions, which act as stress concentration sites. Over extended service periods, PA6 also experiences stress relaxation and creep in hydrogen environments, as molecular chains slowly slide past one another, leading to irreversible deformation. In addition, thermal fluctuations contribute to thermo-mechanical fatigue, which synergizes with mechanical stress to accelerate material aging [57].

To enhance the mechanical performance of PA6 as a liner material in hydrogen storage cylinders, several advanced modification strategies have been developed. One highly effective method is in situ polymerization, which enables nanofillers to disperse uniformly within the PA6 matrix at the molecular level [58,59]. This not only strengthens interfacial bonding but also significantly improves both the strength and toughness of the material. Another widely adopted technique is nanofiller reinforcement, where nanoscale additives such as carbon nanotubes and graphene are introduced into the polymer [60,61,62]. These fillers form a three-dimensional barrier or reinforcement network that restricts molecular mobility, thereby enhancing the material’s mechanical, thermal, and gas barrier properties without compromising its inherent characteristics. The third approach is polymer blending, in which PA6 is combined with other polymers or functional additives to achieve a balanced improvement in toughness and ductility [63,64,65]. This is especially useful in mitigating PA6’s intrinsic brittleness, allowing for better impact resistance under the high-pressure conditions typical of hydrogen storage. These modification strategies—whether applied individually or synergistically—offer a comprehensive pathway to optimize the structural performance of PA6, ensuring it meets the stringent durability and safety requirements of Type IV hydrogen storage applications.

### 5.2. Strategies for Enhancing Mechanical Properties of PA6

#### 5.2.1. In Situ Polymerization of PA6

**Figure 9:**
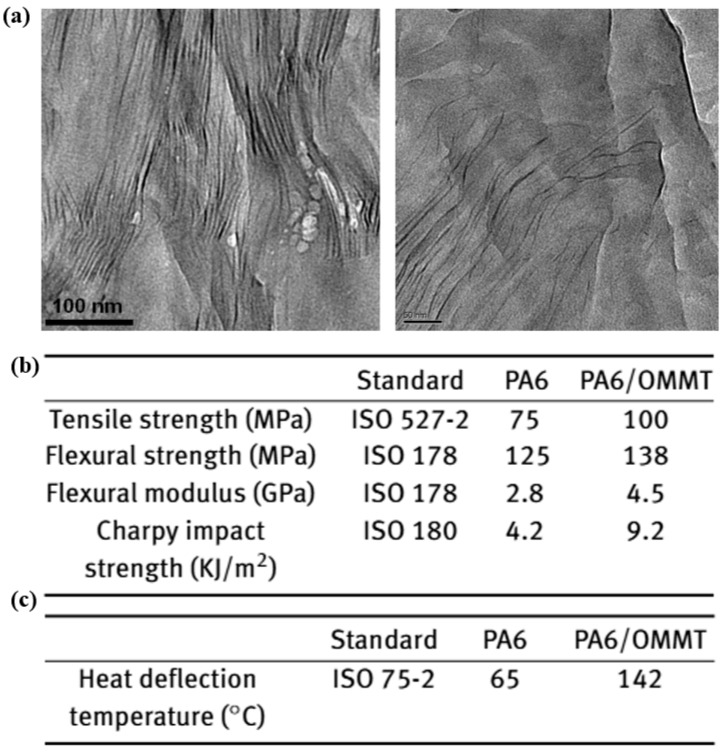
TEM images of PA6 composites with 3.5 wt% MMT (**a**); the mechanical properties (**b**); and heat deflection temperature of PA6 composites [59].

In situ polymerization is a molecular-level modification technique that enables uniform dispersion of nanofillers within polymer matrices, significantly enhancing overall material performance. In the modification of PA6, intercalated polymerization using MMT has attracted considerable attention due to MMT’s layered structure and tunable interfacial properties [59,66,67,68]. The interlayer spacing of MMT modified with cetyltrimethylammonium bromide (CTAB) increased from 1.2 nm to 3.13 nm and further expanded to 10–20 nm during in situ polymerization of PA6, forming an exfoliated nanocomposite (Figure 9a) [59]. A one-step in situ intercalation polymerization process allows the modified MMT to be blended directly with ε-caprolactam monomers for simultaneous ion exchange, monomer intercalation, and polymerization, thereby simplifying the preparation procedure. When a small amount of modified MMT is incorporated into a PA6 via in situ intercalation polymerization, the resulting nanocomposite exhibits significant improvements in mechanical properties and thermal stability (Figure 9b,c). These enhancements are primarily attributed to the nanoscale effects of the MMT layers and their interfacial stress transfer mechanisms. The well-dispersed platelets reinforce the rigidity of PA6 and significantly boost its gas barrier performance—an essential property for the airtightness required in hydrogen storage cylinder liners.

#### 5.2.2. Filler-Reinforced Composite

**Figure 10:**
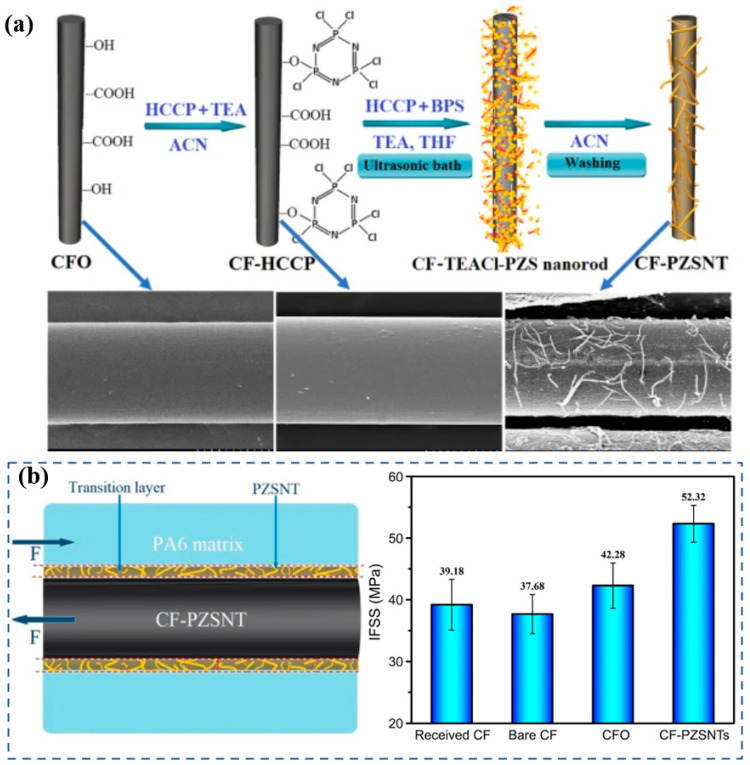
The modification process of fillers (**a**). The interfacial bonding and interfacial shear strength of PA6 composites (**b**) [70].

Nanocarbon materials (such as CNT and graphene) and inorganic reinforcements (like basalt fibers) are considered ideal fillers for enhancing the mechanical and barrier properties of PA6, owing to their exceptional intrinsic strength and nanoscale effects [62,69,70,71]. Compared with neat PA6, the elastic modulus and the yield strength of the composite are greatly improved by about 214% and 162%, respectively, with the incorporation of only 2 wt% CNTs [62]. The dispersion of fillers and their interfacial bonding with the PA6 matrix are critically related to the mechanical reinforcement of PA6 composites. For instance, 3D printing techniques have been employed to promote the oriented alignment of modified fillers within the polymer, thereby enhancing composite performance [69]. Additionally, extensive research has focused on surface modification of fillers to strengthen the interfacial adhesion with the PA6 matrix, achieving more pronounced reinforcement effects. A layer of hierarchical poly(cyclotriphosphazene-co-4,4′-sulfonyldiphonel) (PZS) coating combining with nanotubes (PZSNTs) was assembled uniformly surrounding CF surfaces to enhance the interface adhesion of the CF-reinforced PA6 (CF/PA6) composites, as illustrated in Figure 10a [70]. The transition of the PZSNT coating not only significantly enhances the interfacial wettability and mechanical interlocking between the fibers and PA6 matrix but also provides a loading absorption/transfer layer that could efficiently transfer stress and assist in holding back excessive stress spreading in the flaw and improve overall mechanical properties (Figure 10b). Collectively, these advances contribute to significantly enhanced durability and gas barrier performance of PA6-based composites, supporting their application in high-pressure hydrogen storage environments.

#### 5.2.3. Blending Modification

**Figure 11:**
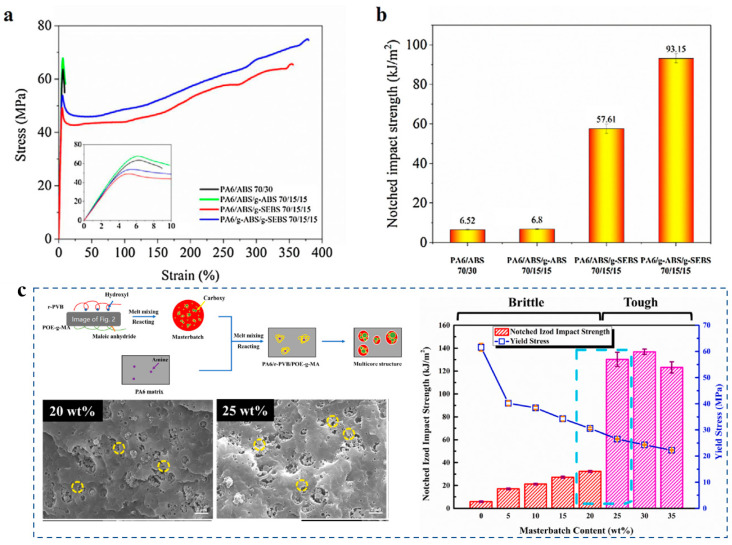
The mechanical properties of PA6/ABS composites (**a**,**b**). The microstructure and impact strength of r-PVB/POE-g-MA/PA6 composites (**c**) [73,76].

As a semi-crystalline polymer, PA6 exhibits brittleness, especially under low temperatures or impact loads. Therefore, toughening modification is a critical challenge in the development of PA6 liners for hydrogen storage cylinders. Blending with elastomers and additives is an effective approach to toughen PA6 [72,73,74,75,76]. Elastomers such as acrylonitrile–butadiene–styrene copolymer (ABS) and poly(ethylene-1-octene) (POE) typically exhibit limited toughening effects due to poor interfacial compatibility with PA6. For instance, Sui et al. reported that PA6/ABS binary blends showed low elongation at break (<25%) and impact strength (6.52 kJ/m^2^) (Figure 11a,b) [73]. However, the introduction of a third-component compatibilizer significantly increases these values to ~350% and 57.61 kJ/m^2^, respectively (Figure 11a,b). The compatibilizer reduces the dispersed phase size and promotes the formation of a unique “soft shell-encapsulated hard core” structure. In another system, reactive melt blending of PA6 with plasticized poly(vinyl butyral) residue (r-PVB)/maleated poly(ethylene-1-octene) (POE-g-MA) masterbatch yields a multicore-structured super-toughened PA6 composite, where POE-g-MA becomes encapsulated by r-PVB within the matrix (Figure 11c) [76]. A brittle-to-ductile transition occurs at a critical interparticle distance of ~0.30 μm, resulting in a 22–24-fold enhancement in impact strength compared to neat PA6 (Figure 11c). This exceptional toughness arises from the combined effects of r-PVB/POE-g-MA flexibility and strong interfacial adhesion among the three components. These synergistic toughening mechanisms, characterized by optimized phase morphology and interfacial adhesion, demonstrate significant potential for developing high-performance PA6-based liners that simultaneously meet the stringent impact resistance and mechanical strength requirements for hydrogen storage cylinder applications.

To comprehensively improve the mechanical properties of PA6, a combination of in situ polymerization, nanofiller reinforcement, or blending strategies is employed. The combination of strategies tends to exert a synergistic effect. In situ polymerization allows for the uniform dispersion of nanofillers (e.g., graphene, carbon nanotubes, or nanoclay) within the PA6 matrix during polymer formation, improving interfacial adhesion and minimizing agglomeration. This method ensures strong bonding between the nanofillers and the PA6 chains, leading to better stress transfer and load distribution. Meanwhile, nanofiller reinforcement provides additional strength and stiffness by acting as nucleation sites for crystallization, which refines the polymer’s microstructure and enhances its tensile and impact properties. Blending PA6 with other polymers or elastomers introduces toughness and ductility, counteracting the potential brittleness caused by nanofillers. The synergy arises when these strategies are integrated: in situ polymerization ensures optimal nanofiller dispersion, nanofillers enhance stiffness and strength, and blending improves fracture resistance, resulting in a balanced combination of high strength, toughness, and durability. This multi-faceted approach leverages the advantages of each method while mitigating their individual limitations, creating a high-performance material suitable for demanding engineering applications.

## 6. Conclusions

PA6 has emerged as a highly promising liner material for Type IV hydrogen storage cylinders, demonstrating an exceptional combination of mechanical robustness, thermal stability, and hydrogen barrier capabilities. The material’s semi-crystalline structure and polar amide groups provide inherent advantages for high-pressure hydrogen containment, though challenges persist in achieving optimal performance under extreme operating conditions. Recent advances in material science have revealed the transformative potential of nanoscale modifications and advanced processing techniques in overcoming these limitations. The integration of graphene, carbon nanotubes, and layered silicates into PA6 matrices has proven particularly effective in creating tortuous diffusion pathways that dramatically reduce hydrogen permeability. Simultaneously, innovative approaches such as in situ polymerization and reactive compatibilization have successfully addressed the traditional trade-off between barrier properties and mechanical toughness. These developments have yielded materials capable of withstanding the rigorous demands of hydrogen storage applications while maintaining structural integrity over extended service periods.

Current research on PA6 liners for hydrogen storage still faces several critical limitations that hinder commercial deployment. A major gap exists in standardized testing protocols, as current methods fail to adequately replicate real-world conditions involving simultaneous thermal cycling (−40 to 85 °C), mechanical loading (35–100 MPa), and hydrogen permeation under dynamic pressure variations. The field also lacks integrated simulation approaches that can bridge molecular-scale interface interactions with macroscopic structural performance predictions. Furthermore, comprehensive lifecycle assessments are notably absent, particularly regarding long-term environmental impacts and the recyclability of nanocomposite materials. These shortcomings result in significant performance prediction uncertainties, with pilot studies showing discrepancies between laboratory and field performance data.

Future research should focus on establishing standardized multiaxial testing protocols, developing integrated multiscale simulation frameworks, and conducting comprehensive lifecycle assessments to advance PA6-based hydrogen storage liners toward commercial viability. Furthermore, the path toward commercial viability requires continued innovation in material design and processing methodologies. The development of multifunctional nanocomposites, optimization of manufacturing scalability, and establishment of comprehensive performance evaluation standards will be critical to realizing the full potential of PA6-based liners. As the hydrogen economy continues to expand, these advanced polymer composites are poised to play a pivotal role in enabling safe, efficient, and sustainable hydrogen storage solutions. The convergence of fundamental research and industrial application will undoubtedly drive further breakthroughs in this field, contributing significantly to the global transition toward clean energy systems. This comprehensive exploration of PA6’s capabilities and limitations underscores its vital position in the future of hydrogen storage technology while highlighting the exciting opportunities for further material optimization and system integration. The collective insights presented in this review provide both a scientific foundation and practical guidance for advancing polymer liner technologies to meet the evolving demands of the hydrogen energy sector.
